# Die Pandemie offenbart die Defizite des transnationalen Menschenrechtsschutzes: Ein Plädoyer für eine Konfliktperspektive auf globale Lieferketten

**DOI:** 10.1007/s42597-020-00052-y

**Published:** 2020-11-19

**Authors:** Christian Scheper, Carolina A. Vestena

**Affiliations:** Institut für Entwicklung und Frieden, Universität Duisburg-Essen, Duisburg, Deutschland

**Keywords:** Lieferketten, Friedens- und Konfliktforschung, Menschenrechte, Arbeitsrechte, Transnationale Governance, Soziale Standards, Supply chains, Peace and conflict research, Human rights, Labour rights, Transnational governance, Social standards

## Abstract

Die Corona-Pandemie hat zu massiven Disruptionen globaler Lieferketten geführt und damit die Exportökonomien vieler Produktionsländer existenziell getroffen. Besonders dramatisch ist dies für die Arbeiter:innen am Anfang der Wertschöpfungskette. Die Verletzungen international anerkannter sozialer und ökonomischer Rechte in globalen Lieferketten sind heute massiver als vor der Krise und offenbaren ein weitreichendes Konfliktpotenzial. Der Beitrag verdeutlicht diese Situation beispielhaft für Brasilien und Indien und problematisiert auf dieser Grundlage globale Lieferketten als Thema der Friedens- und Konfliktforschung. Lieferketten sind Räume politischer Auseinandersetzungen um materielle und ideologische Bedingungen der Produktion, die durch eine Vielzahl institutioneller Kontexte und Akteure mitgestaltet werden. Ein umkämpfter Gegenstand ist dabei der mangelnde Arbeits- und Menschenrechtsschutz. Der dominante Weg zur Adressierung dieses Mangels sind die Institutionen und Praktiken der transnationalen privaten Governance durch Standards, Zertifizierung, Monitoring und Audits. In der Pandemie offenbaren diese ohnehin lückenhaften Ansätze, dass sie gerade in Krisenzeiten ungeeignet sind, um grundlegende Arbeits- und Menschenrechte zu sichern und bestehende Konflikte zu befrieden. Der Beitrag betont abschließend die Verbindung zwischen globalen Lieferketten und lokalen Konflikten und plädiert für eine Forschungsagenda zu „Lieferketten und Konflikt“.

## Abstract (en)

The corona pandemic has led to a massive disruption of global supply chains and has thus existentially affected the export economies of many producing countries. This is particularly dramatic for workers at the beginning of the value chain. The violations of internationally recognized social and economic rights in global supply chains are more massive today than before the crisis and reveal a far-reaching potential for new conflicts. The article illustrates this situation in the case of Brazil and India and, on this basis, raises the issue of global supply chains as a topic for peace and conflict research. Supply chains are spaces of political conflict about material and ideological conditions of production, which are shaped by a variety of institutional contexts and actors. One of the most controversial issues is the lack of labour and human rights protection. The dominant way to address this lack is through institutions and practices of transnational private governance—standards, certification, monitoring and audits. In the pandemic, these already patchy approaches reveal that, especially in times of crisis, they are unsuitable for securing basic labour and human rights and for pacifying existing conflicts. The article concludes with an emphasis of the linkages between global supply chains and local conflicts and argues for an extended research agenda on “supply chains and conflict”.

## Einleitung

Die Corona-Pandemie hat zu massiven Disruptionen globaler Lieferketten geführt. Von einer anfänglichen Importkrise durch Einschränkungen in China über den Einbruch der Nachfrage in Konsumländern und anschließenden staatlich verordneten Lockdowns wurden die Exportökonomien vieler Produktionsländer von der Pandemie existenziell getroffen. Zahlreiche Betriebe mussten ihre Produktion herunterfahren oder schließen. Am schwersten sind davon Arbeiter:innen am Anfang der Wertschöpfungskette getroffen, häufig ohne Kündigungsschutz, vielfach informell beschäftigt.

Die Verletzungen grundlegender sozialer und ökonomischer Rechte in globalen Lieferketten sind während der Pandemiekrise offensichtlich und haben weitreichende Folgen für soziale Konflikte. In diesem Beitrag nehmen wir diese Zäsur globaler Produktions- und Handelsbeziehungen zum Anlass, um globale Lieferketten aus einer Friedens- und Konfliktforschungsperspektive zu betrachten. Im Mittelpunkt der Disziplin stehen häufig gewalttätige bzw. militärische Disruptionen bzw. ihre Vermeidung. Konflikte und Konfliktregionen dienen auch als Ausgangspunkt für die Untersuchung sozio-ökonomischer Verhältnisse, die teilweise als Ursache oder relevante Dimension des Konflikts mit berücksichtigt werden (vgl. etwa Silve 2018).^1^ Die staatliche Kapazität steht hierbei meist im Vordergrund (Silve 2018:1511), weniger aber die transnationale Dimension wirtschaftlicher Verflechtung. Eine breitere Rezeption des Zusammenhangs zwischen globalen Lieferketten, ihrer politischen Gestaltung und gesellschaftlichen Konflikten findet bisher außerhalb der Friedens- und Konfliktforschung statt (vgl. etwa Cowen 2014).

Die Lieferketten-Debatte, die vor allem im Kontext der Betriebswirtschaftslehre, der Wirtschaftssoziologie oder Geographie behandelt wird, berücksichtigt wiederum kaum weitergehende gesellschaftliche Konflikte. Wie Levy (2008, S. 951) betont, betrachtet die Governance-Perspektive auf Lieferketten vor allem Formen der ökonomischen Koordination, weniger Formen der politischen Auseinandersetzung als Grundlage und Folge solcher Koordinationsprozesse. Die institutionelle und diskursive Einbettung der Märkte und Produktionsnetzwerke bedarf daher weiterer Untersuchung aus einer politischen Perspektive, die für gesellschaftliche Konflikte sensibel ist. Denn diese Einbettung ist Grundpfeiler des Arbeits- und Menschenrechtsschutzes, aber durchaus stets konflikthaft. Dies zeigt sich besonders deutlich in den Möglichkeiten der Konfliktaustragung, weniger in „von oben“ gesetzten Mindeststandards: in kollektiven Organisationsformen der Arbeiter:innen, ihrer gesetzlichen Ermöglichung und Einhegung, in der Artikulation transnationaler und lokaler NGOs und Gewerkschaften oder auch im Verlauf offener Arbeitsrechtskonflikte, wie Streiks und Fabrikbesetzungen, die nicht selten mit staatlicher Gewalt beantwortet werden.

Während „Arbeitskämpfe“ auch durchaus allgemein als Aspekt der Friedens- und Konfliktforschung gesehen werden (Kißler 2010), so erfordert die transnationale Dimension der Arbeitsteilung und -organisation ein eigenes Konfliktkapitel, das bisher nur schemenhaft geschrieben wurde. Angesichts ihrer stets umkämpften und durch starke politisch-ökonomische Machtungleichgewichte geprägten Beziehungen ist die Bedeutung von Lieferketten für die internationale Friedens- und Konfliktforschung hoch, aber gerade aus Sicht der konsumorientierten OECD-Länder häufig nicht offensichtlich. Es ist die Pandemie, die uns deutlicher denn je zeigt, dass globale Strukturen und Praktiken der Produktion mit Formen der Gewalt und des Konflikts untrennbar zusammenhängen. Dort, wo Frieden in einem positiven Sinne verstanden wird, also umfassend soziale und institutionelle Formen innergesellschaftlicher Gewalt mitgedacht werden, dort müssen diese Gewaltformen, ihre Strukturen und Praktiken auch zum Bestandteil der Analyse werden.

In unserem Beitrag erläutern wir diese Behauptung, indem wir zunächst globale Lieferketten konzeptionell als umkämpfte politische Räume fassen (Abschn. 2). Derzeitige nationale und internationale Bemühungen um extraterritorial gültige menschenrechtliche Sorgfaltspflichten in der Lieferkette identifizieren wir hierbei als aktuelles empirisches Moment der politisch-normativen Umstrittenheit von Lieferkettenbeziehungen. Es entstand damit ein neues transnationales Terrain für (stets vorhandene) Kämpfe um Arbeits- und Menschenrechte. Es ist aber fluid und Gegenstand gesellschaftlicher Auseinandersetzungen, daher prozesshaft zu verstehen. Besetzt wird es bisher einseitig durch private und hybride Governance-Praktiken auf Basis des Managements globaler Leitunternehmen in der Lieferkette. Die Disruptionen durch die Pandemie, die den Mangel des Arbeits- und Menschenrechtsschutzes offenlegen und verschärfen, zeigen besonders deutlich die fatalen Folgen dieser Schieflage. Dies thematisieren wir in Abschn. 3 mit Blick auf national und transnational geprägte Pandemie-Konflikte in Indien und Brasilien. Beide Länder sind stark von der Pandemie betroffen und beide Regierungen antworten mit starken Einschränkungen von Arbeits- und Menschenrechten, teilweise begleitet von Gewalt gegen Protestbewegungen. Wir schließen mit einem Plädoyer für eine erweiterte Forschungsagenda zu globalen Lieferketten und Konfliktforschung.

## Globale Lieferketten als Macht- und Konflikträume

Globale Lieferketten^2^ sind heute die dominante Form der Organisation von Investition, Produktion und Handel in der globalen politischen Ökonomie (ILO 2016). Etwa die Hälfte aller internationalen Handelsströme ist inzwischen auf transnationale Lieferketten zurückzuführen (World Bank 2020). Damit geht die Auseinandersetzung mit Lieferketten auch weit über die ursprünglich betriebswirtschaftliche Perspektive hinaus. Politisch wurde ihre Bedeutung bereits früh durch weltsystemtheoretische Betrachtungen internationaler Arbeitsteilung deutlich (Hopkins und Wallerstein 1994). Heute begründen Lieferketten eine umfassende interdisziplinäre Forschungslandschaft (Gibbon et al. 2008). Wichtig für die Betrachtung der konfliktiven Auswirkungen der Pandemie erscheint uns zunächst, Lieferketten als ein politisches und damit konfliktbehaftetes Feld zu verstehen, ein Aspekt, der in der erwähnten Lieferketten-Forschung eher wenig beleuchtet wurde.^3^ Levy (2008) zeigt im Rückgriff auf die Theorie Globaler Produktionsnetzwerke, die institutionalistische Management-Theorie und neogramscianische Ansätze der Internationalen Politischen Ökonomie, dass Lieferketten sowohl materielle als auch ideologische Machtdimensionen aufweisen und durch Kämpfe um Deutungsmacht geprägt sind. Sie sind kein neutrales wirtschaftliches Terrain, sondern (re)produzieren bestehende soziale Verhältnisse und Widersprüche. Lieferketten haben letztlich die betrieblichen Konflikte der kapitalistischen Industrieproduktion auf ein transnationales Terrain verlagert. Ihre Strukturen und Praktiken sind transnational, aber durch multiple nationale Rahmenbedingungen institutionell sehr unterschiedlich eingebettet. Sie sind dabei durchweg geprägt von Arbeitskonflikten unter Bedingungen nationaler und transnationaler Ungleichheit, einschließlich der vielfältigen rassistischen, klassistischen, sexistischen, kolonialistischen und sonstigen intersektional diskriminierenden Ausschlüsse, die in kapitalistischen Gesellschaften fortbestehen (Levy 2008, S. 945). Erst diese erklären letztlich die Profitabilität der globalen Organisation von Lieferketten, denn diese basiert zu einem wesentlichen Teil auf der betriebswirtschaftlichen *Ausnutzung* institutioneller Unterschiede sowie gesellschaftlicher Ungleichheiten in transnationaler Perspektive (Bair und Werner 2011; Bonacich et al. 2008; Ferus-Comelo 2006; Tsing 2009). Es ist kein Zufall, dass die arbeitsintensivsten und am schlechtesten bezahlten Tätigkeiten am Anfang der Lieferkette häufig von jungen Frauen mit Migrationserfahrung in Ländern mit schwachem Arbeitsschutz und mangelnden Gewerkschaftsfreiheiten ausgeübt werden.

Somit befördern Lieferketten eine spezifische internationale Arbeitsteilung, die Warenproduktion in der Regel auf Regionen des Globalen Südens verlagert und höheren Profit für Leitunternehmen aufgrund struktureller Abhängigkeitsverhältnisse und Ungleichheiten ermöglicht (Graf et al. 2020). Die damit erreichten Entwicklungsfortschritte sind in produzierenden Ländern teilweise erheblich, gerade auch für Frauen und andere benachteiligte Bevölkerungsgruppen (World Bank 2020). Sie führen aber gleichzeitig zur Reproduktion ebenjener Ungleichheitsstrukturen und Diskriminierungspraktiken. Die Kosten und die Bedingungen der Arbeit, die immer konfliktbehaftet sind (Kißler 2010), werden damit keineswegs von gleichgewichtigen Positionen aus verhandelt. Arbeiter:innen, häufig mit Migrationserfahrungen oder gar an die Erwerbsarbeit gekoppeltem Aufenthaltsstatus, müssen die vorhandenen Bedingungen häufig akzeptieren, um ein Einkommen generieren zu können (Fischer 2020). Das Konfrontationsfeld der Lieferketten konstituiert sich dabei durch multiple Akteure: Unternehmen, Staaten, internationale und zivilgesellschaftliche Organisationen, Beratungsfirmen, Gewerkschaften, Verbände und Arbeiter:innen (Levy 2008).

### Transnationale Governance zur Befriedung konfliktiver Beziehungen in Lieferketten?

Während sich aus deutscher Sicht innerhalb nationaler industrieller Beziehungen die Wege und Strategien zur Befriedung von Arbeitsrechtskonflikten historisch stark gefestigt haben (Tarifautonomie, Sozialpartnerschaft, Arbeitsschutzgesetze, usw.), sind sie in vielen Ländern viel stärker umstritten. Transnational stehen sie erst recht weitgehend zur Disposition und ihre gesellschaftlichen Aus- und Wechselwirkungen sind vielfältiger. Hier spiegeln Konflikte um Arbeitsrechte wesentlich die durch Levy (2008) aufgezeigten hegemonialen Deutungskämpfe und Machtverhältnisse wider. Nehmen wir die globale Textil- und Bekleidungsproduktion als Beispiel, so folgt hier die Governance grundlegender Standards den weitgehend käufergetriebenen Wertschöpfungsstrukturen. Arbeitsstandards und -rechte sind zwar national gesetzlich geregelt, werden aber hinsichtlich ihres normativen Gehalts sowie ihrer Anreiz- und Durchsetzungsmechanismen wesentlich „vom Ende“ der Lieferkette aus regiert, insbesondere durch Corporate Governance im Rahmen privatrechtlicher Lieferverträge (vgl. Cutler und Dietz 2017) und private Überwachungsmechanismen.

Hierbei beziehen sich Unternehmen normativ aber auch zunehmend auf internationale Arbeits- und Menschenrechte (Scheper 2019), etwa im Kontext globaler Rahmenabkommen mit Gewerkschaftsverbänden (Fichter et al. 2011) sowie hybrider transnationaler Steuerungsinstrumente, wie industrieller Mindeststandards, Zertifizierungen, Monitoring und Auditing (Bartley 2018; LeBaron und Lister 2015; Locke 2013). Letztere sind dabei immer wieder auch Gegenstand transnationaler zivilgesellschaftlicher Arbeitsrechtskämpfe (vgl. etwa Zajak et al. 2017). Analytisch sinnvoll ist daher die enge Verknüpfung transnationaler Lieferkettenstrukturen mit der Analyse politischer Arbeitskonflikte und der jeweiligen nationalen Praktiken und Institutionen zu ihrer Befriedung oder Einhegung.

### Menschenrechtliche Sorgfaltspflicht als Konzept zur Sicherung sozialer Mindeststandards

Angesichts ihres wirtschaftlichen Einflusses orientiert sich auch die Auseinandersetzung mit ethischer Governance stark an den transnationalen Leitunternehmen. Heute wird die soziale und ökologische Verantwortung in der Lieferkette von nahezu allen großen Konzernen als Kernaspekt der *Corporate Responsibility* verstanden. Es haben sich auf dieser Grundlage umfassende Praktiken, Institutionen und sogar eigene Märkte des ethischen Lieferketten-Managements etabliert (LeBaron et al. 2017; LeBaron und Lister 2015; Scheper 2018). Wir können diese Entwicklung zu einem gewissen Grad als Zugeständnisse angesichts der zunehmenden gesellschaftlichen Bedeutung globaler Lieferketten, ihrer inhärenten Arbeits- und Rechtskonflikte und der daraus resultierenden Krise der profitablen Positionen einflussreicher Unternehmen verstehen (vgl. Sum 2010).

Die jüngste Entwicklung in diesem Verantwortungsdiskurs besteht in der Bezugnahme auf internationale Menschenrechte und der Etablierung des Konzepts der menschenrechtlichen Sorgfaltspflicht transnationaler Unternehmen. Normativ ist sie vor allem durch die Vereinten Nationen und die im Menschenrechtsrat etablierten Leitprinzipien für Wirtschaft und Menschenrechte (United Nations 2011) angeleitet. Die menschenrechtliche Sorgfaltspflicht repräsentiert eine Verschiebung der sozialen Konfliktlinien innerhalb globaler Lieferketten. Einerseits begegnen sich Unternehmen weiterhin unter gegenseitiger Konkurrenz und gestalten ihre Produktion entlang ihrer Profitmaxime, was mit einer konsequenten Einhaltung von Menschenrechtsstandards häufig nicht vereinbar ist. Allerdings verlangt der Sorgfaltsdiskurs durchaus das Bekenntnis zu allen völkerrechtlich anerkannten Menschenrechten, die Einführung grundlegender *Policies* des ethischen Lieferkettenmanagements sowie ihre regelmäßige Überprüfung und Wirkungsmessung (vgl. Grabosch und Scheper 2015).

Die Sorgfaltspflicht als ethisches Steuerungskonzept verdeutlicht die Relevanz der von uns vertretenen prozessorientierten Konfliktperspektive auf Lieferketten. Dabei geht es hierbei gerade nicht primär um die Etablierung neuer Mindeststandards, sondern um eine potenzielle Veränderung in der Bearbeitung von politisch-ökonomischen Konflikten durch eine neue rechtlich-institutionelle Einbettung entlang transnationaler Wirtschaftsbeziehungen. Einerseits befürchten kritische Stimmen, dass diese Verschiebung eher als eine Form der hegemonialen Krisenanpassung zu sehen ist, die bisher kaum neue institutionelle Möglichkeiten für Rechteinhaber:innen etabliert hat, um Menschenrechte im Konfliktfall *gegen *transnationale Leitunternehmen durchsetzen zu können (vgl. Deva 2013; Scheper 2019). Andererseits stellt die Etablierung der Sorgfaltspflicht aber zumindest eine Folie für die Einforderung menschenrechtsorientierter Bedingungen entlang der Lieferketten bereit (LeBaron et al. 2017). In diesem Prozess können sich neue Räume für die Organisierung von Arbeiter:innen und ihre transnationalen Unterstützungsnetzwerke öffnen, die sich auch in neuen rechtlichen Institutionen bzw. entsprechenden Forderungen gegenüber beteiligten Regierungen zur Etablierung effektiverer Maßnahmen zum Rechtsschutz verfestigen könnten (ECCHR 2020; Zajak und Scheper 2019; Zajak et al. 2017).

Wir können somit festhalten, dass Lieferketten als umkämpfte Räume zu verstehen sind und dabei grundlegende ethisch-politische Konflikte um Arbeitsbeziehungen heute entlang der Prämissen transnationaler privater und hybrider *Governance* und der internationalen Menschenrechte verhandelt werden. Diese sind zu einem wesentlichen Gestaltungsaspekt geworden, der für die Betrachtung gesellschaftlicher Konflikte notwendig ist, insbesondere in exportorientierten Ländern des Globalen Südens. Dies macht die Pandemie besonders deutlich, was wir im Folgenden am Beispiel der zunehmenden Konflikte entlang des Textilsektors mit Beispielen aus Indien und Brasilien zeigen.

## Auswirkungen der Covid-19 Pandemie auf Lieferketten: Multiple Dimensionen des Konflikts

Als die WHO Covid-19 zur Pandemie erklärte, führten Staaten in der ganzen Welt Isolationsmaßnahmen durch, einschließlich strikter *Lockdowns*. Schnell wurde deutlich, dass auch die transnationalen Warenflüsse in Lieferketten durch die Krise stark beeinträchtigt werden. Spätestens seit Mai 2020 machte die Situation wichtiger Exportländer wie Indien und Brasilien daher weltweite Schlagzeilen. Beide Länder verzeichneten nach den USA die höchsten Infektionszahlen und standen vor massiven sozialen und ökonomischen Herausforderungen.

Zwar weisen Brasilien und Indien diversifizierte Wirtschaften und Produktionssektoren auf, doch hatte der Zusammenbruch des globalen Warenflusses heftige Auswirkungen. Er betrifft vor allem Sektoren, die stark vom globalen Markt abhängig sind, wie den Textilsektor. Beide Länder stellen wichtige Rohstoffe (z. B. Baumwollfaser und Garn) und fertige Produkte (z. B. Stoffe, Bekleidungsstücke) für die globale Textillieferkette her. Die Krise verstärkt den damit verbundenen Druck, „wettbewerbsfähig“ zu bleiben, d. h. vor allem den etablierten Normen attraktiver Investitionsbedingungen zu folgen. Die Folge ist, dass Arbeitsbedingungen und die Möglichkeit der kollektiven Organisierung von Arbeiter:innen in beiden Ländern massiv unter Druck geraten sind. Die bereits ungenügenden Arbeitsbedingungen haben sich im Verlauf der Covid-19-Krise deutlich verschlechtert. Dies hat weitergehende Folgen für die Entwicklung nationaler und transnationaler Arbeitsrechtskonflikte.

### Indien

Indien erreichte im Jahr 2018 weltweit das zweitgrößte Exportvolumen für Textilien im Allgemeinen und das fünftgrößte für Bekleidung. Insgesamt beschäftigt die textile Lieferkette etwa 45 Mio. Menschen im Land und ist damit die drittgrößte Arbeitgeberin nach der Landwirtschaft und dem Bausektor. Insbesondere innerstaatliche Wanderarbeiter:innen sind in der Textilindustrie aktiv und der Anteil an Frauen steigt zunehmend (Fair Wear Foundation 2019). Die Verarbeitung fertiger Textilwaren verteilt sich in verschiedenen industriellen Drehkreuzen über das Land. In diesen herrschen ganz unterschiedliche Rahmenbedingungen, aber zugleich eine fast durchgehend mangelhafte Einhaltung sozialer Standards und Arbeitsrechte (Asia Floor Wage Alliance 2020a).

Die mit nur vier Stunden Vorlauf angekündigten Lockdown-Maßnahmen der indischen Regierung, die ab dem 24. März 2020 zur Schließung von Fabriken, Geschäften und großen Teilen des öffentlichen Lebens führten, hatten dramatische Folgen. Viele Menschen konnten keine Lebensmittel mehr einkaufen und es kam zu teils chaotischen Migrationsbewegungen. Da kaum noch Arbeit in den Hauptstädten zu finden war, mussten Arbeiter:innen, die häufig als Tagelöhner beschäftigt sind, ihr Überleben in ihren Heimatdörfern sichern. Viele Millionen Wanderarbeiter:innen begaben sich zu Fuß auf den Heimweg in teils hunderte Kilometer weit entfernte Dörfer. Sie waren vielfach mit Gewalt konfrontiert, sowohl durch die Polizei, als auch durch Teile der Bevölkerung, die, angetrieben durch rassistische und klassistische Vorurteile, die Wanderarbeiter:innen als „Virenverbreiter“ brandmarkten. So wurden sie teils aus den eigenen Dörfern vertrieben oder dort isoliert (vgl. Asia Floor Wage Alliance 2020b).

Die Migrationsbewegungen trafen wiederum die Textilindustrie beträchtlich, da diese stark von spezialisierten Wanderarbeiter:innen abhängig ist. Seit Ende des *Lockdowns* können Unternehmen diese Arbeitskräfte nur schwer zurückgewinnen. Die Wiederaufnahme der Produktion erfolgte auch nur selten unter Einhaltung notwendiger Schutzmaßnahmen, insbesondere für Risikogruppen und Frauen (Asia Floor Wage Alliance 2020a). Nicht nur die Unkontrollierbarkeit des Infektionsgeschehens stellt ein unsicheres Umfeld für die Arbeiter:innen dar. Das bereits mangelhafte soziale Sicherheitsnetz wird weiter dadurch geschwächt, dass diverse Landesregierungen grundlegende Arbeitsrechte auf unbestimmte Zeit ausgesetzt haben (Fair Labor Association 2020). Arbeitszeiten bis 12 Stunden täglich und 72 Stunden wöchentlich, die verstärkte Unterdrückung von Gewerkschaftsarbeit, kollektiver Organisierung und Streiks sowie das Aussetzen von Mindestlöhnen sind nun vielfach möglich (Business and Human Rights Resource Centre 2020). Kinder- und Zwangsarbeit haben aufgrund des Mangels an Arbeitskräften wieder zugenommen.

Arbeitsrechtskonflikte werden in Indien derzeit unter Rückgriff auf hegemoniale Diskurse des Wettbewerbsstaats im Kontext globaler Produktions- und Lieferkettenstrukturen massiv verschärft. Hierbei ergänzen sich transnationale unternehmerische Einkaufspraktiken und dadurch verursachter betriebswirtschaftlicher Druck mit staatlichen Institutionen die den Rechtsschutz gezielt einschränken. Dies ruft wiederum Proteste durch Arbeiter:innen hervor, die teils gewaltsam beendet oder unterdrückt werden.

### Brasilien

Die brasilianische Bekleidungsindustrie hat zwar in den letzten Jahren auf dem globalen Markt etwas an Bedeutung verloren, allerdings ist die Textilindustrie noch immer hoch relevant, auch für den Export von Rohstoffen (ABIT 2019). Die unterschiedlichen Teile der textilen Lieferkette umfassen etwa 8 Mio. Beschäftige im Land. Frauen machten 2019 75 % der Arbeitskraft aus. Einem Bericht der Brasilianischen Assoziation der Textilindustrie (ABIT) zufolge erhalten junge Erwachsene und Wanderarbeiter:innen aus anderen Ländern Südamerikas häufig die erste Beschäftigung im Textilsektor (ABIT 2019). Internationale arbeitsrechtliche Standards werden auch in Brasilien in der Produktion häufig missachtet und die übliche Praxis der Unterauftragsvergabe erschwert maßgeblich ein zuverlässiges Monitoring der gesamten Lieferkette (Bignami 2017).

Die Pandemie verschärft diese Problemlagen, insbesondere hinsichtlich der sanitären Bedingungen und der Entlohnung. Die Arbeitslosigkeit erreichte im Juli 2020 die historische Höchstgrenze von 47 % der erwerbsfähigen Bevölkerung (IBGE 2020); im Textilsektor fielen ca. 20 % der Arbeitsplätze weg (Mariano 2020). Die Regierung führte keine spezifischen Maßnahmen für den Textilsektor durch, allerdings wurden, wie in Indien, grundlegende Arbeitsrechte ausgesetzt. Arbeiter:innen mussten während der Fabrikschließungen Erholungsurlaub in Anspruch nehmen und die staatliche soziale Sicherung wurde per Dekret im März und April 2020 suspendiert (Fair Labor Association 2020). Auf Grundlage einer Umfrage mit Unternehmen im Textilsektor berichtet die ABIT, dass 62 % der Befragten zunächst auf Zwangsurlaub als Kostenanpassungsmaßnahme zurückgriffen, dennoch im Anschluss Entlassungen nicht mehr vermeiden konnten (ABIT 2020). Viele der Entlassenen sind Wanderarbeiter:innen und Frauen, die um eine Weiterbeschäftigung ringen, um ihren Lebensunterhalt weiter finanzieren zu können (Mantovani 2020).

Auch in Brasilien steht der zunehmende Druck auf Arbeiter:innen in einem unmittelbaren Zusammenhang mit der wirkmächtigen Vorstellung transnationaler Wettbewerbsfähigkeit in Lieferkettenstrukturen. Die brasilianische Regierung wird seit 2019 zunehmend autoritärer (Vestena 2020). Der Schutz fundamentaler Prinzipien und Rechte, wie Transparenz und Teilhabe der Arbeiter:innen, wird weitgehend missachtet. Gewerkschaften und andere zivilgesellschaftliche Akteure reagierten nur schwach auf die Krise. Allerdings wurden ihre Konfrontationen mit der Regierung stets mit der Androhung von Gewalt beantwortet.

Die Verschärfung solcher lokalen Konflikte, auch im Bereich der sozioökonomischen Verhältnisse, wiederholt sich in ähnlicher Weise in unterschiedlichen Lieferketten des Landes. Die transnationale Komponente des Arbeitsrechtskonflikts, also zivilgesellschaftliche und gewerkschaftliche Netzwerke und Multiakteurs-Organisationen, scheint in der Krise weniger sichtbar geworden zu sein. Einseitige Einschränkungen seitens der Regierung bestimmen derzeit die Entwicklung und konterkarieren jahrelange nationale und transnationale Bemühungen zum Arbeitsrechtsschutz.

## Fazit

Die beiden Beispiele Indien und Brasilien zeigen verschiedene Auswirkungsdimensionen der Pandemie sowohl unmittelbar auf die Produktionsbedingungen entlang globaler Lieferketten als auch auf den regulatorischen Rahmen, in dem sich zivilgesellschaftliche Organisationen sowie Arbeiter:innen und Gewerkschaften organisieren. In der Pandemie erfolgen in beiden Ländern einseitige Verschiebungen der Regulierung von Arbeitsrechten zulasten der Rechteinhaber:innen. In beiden Ländern vermischt sich die damit verbundene soziale Katastrophe mit einem Diskurs des Wettbewerbszwangs sowie zunehmend autoritären Strukturen, Gewalt oder zumindest Gewalttendenzen. In Indien betrifft dies vor allem Gewalt gegen Wanderarbeiter:innen, die bereits stark marginalisierten Bevölkerungsgruppen angehören, und in Brasilien beobachten wir Militarisierungstendenzen der Regierung, die unter anderem mit weiteren Einschränkungen für Arbeitnehmer:innen und Gewerkschaftsrechten einhergehen können.

Globale Lieferketten fungieren in beiden Fällen als Terrain, auf dem sozio-ökonomische Konflikte hervorgerufen, verwaltet oder eingehegt werden, da Akteure stets um einen ungestörten Warenfluss bemüht sind. Vorherrschende Diskurse internationaler Wettbewerbsfähigkeit schreiben sich wirkmächtig in die nationalen Positionen bestehender Arbeitsrechtskonflikte ein. Brechen ihre Grundlagen und die Handlungsmöglichkeiten transnationaler Konfliktparteien wie in der Covid-19-Krise ein, so zeigen sich die brutalen Abhängigkeiten des Menschenrechtsschutzes von eben diesen Steuerungs- und Konfliktbearbeitungsmechanismen, in denen der Staat seiner ordnungspolitischen Funktion nur mangelhaft nachkommt. Sein Handeln richtet er dabei vor allem am Wiederaufleben des Warenflusses und dominanten Erzählungen der Wettbewerbsfähigkeit aus, zulasten von Arbeitsschutzgesetzen und Gewerkschaftsfreiheiten. Mit der Pandemie sind bestehende Konflikte in globalen Lieferketten also nicht nur offensichtlicher geworden, sondern haben sich vielfach auch verschärft.

Die unternehmensorientierten Prämissen privater transnationaler Governance, die vor allem auf Mindeststandards, nicht aber auf ermächtigende Mechanismen zur Bearbeitung bestehender Arbeitskonflikte abzielen, erweisen sich in dieser Krise als besonders ungeeignet. Die über Jahre hinweg erkämpften sozialen Standards, die Unternehmen in Lieferketten weltweit verpflichten, dienen weiterhin als Folie für kontinuierliche Auseinandersetzungen der politisch engagierten Akteur:innen, insbesondere im Globalen Süden. Während der Krise haben sie aber kaum zum Menschenrechtsschutz beigetragen, denn funktionierende transnationale Lieferbeziehungen sind zu ihrer Voraussetzung geworden. Die Disruptionen der Lieferbeziehungen haben Mechanismen des Schutzes, aber auch des politischen Streits um soziale und ökonomische Rechte durch Arbeiter:innen- und Multiakteurs-Netzwerke gestört. Die Pandemie wird zum Brennglas: Die sozialen Konflikte intensivieren sich und werden offengelegt und gleichzeitig scheitern die noch immer weitgehend auf private Vertragsbeziehungen bauenden Mechanismen zu ihrer Bearbeitung mehr denn je. Der Verlauf der Pandemie zeigt, dass eine Stabilisierung der Lieferketten vermutlich noch in weiter Ferne liegt und eine Erholung der Produktionsländer von der Krise längere Zeit in Anspruch nehmen wird. Es ist daher eher mit einer Zunahme der sozioökonomisch bedingten Konflikte zu rechnen. Die Perspektive der Konfliktforschung auf Lieferketten und ihre transnationale Verflechtung wird hier verstärkt notwendig sein.

Wir können auf dieser Grundlage erste Kontouren für eine Forschungsagenda zu transnationalen Lieferketten und Konflikt darlegen. Erstens würde eine solche Agenda die politische, umkämpfte Dimension globaler Lieferketten herausstellen, die stets transnational und national bzw. lokal eingebettet ist. Die Friedens- und Konfliktforschung kann vertiefend auf Konfliktpotenziale eingehen und dazu beitragen, dass die Analyse globaler Lieferketten die multiplen gesellschaftlichen Herausforderungen und sozialen Konflikte durch Machtungleichgewichte und fehlenden Rechtsschutz nicht nur als lokale Rahmenbedingungen und Defizite der Produktionsländer, sondern als Element und Ausdruck transnationaler Wertschöpfungsbeziehungen versteht. Die transnationale Regulierung auf Basis privater und hybrider Governance ist dabei Raum und Ausdruck kollektiver Auseinandersetzungen um normative Deutungsmacht, der Erfassung und Verteilung ökonomischer Wertschöpfung und ihrer ideologischen wie materiellen Bedingungen.

Zweitens könnte eine Betrachtung der Konfliktdimensionen sowohl aktuelle regressiven Prozesse des Rechtsentzugs in den Blick nehmen, als auch die weit verbreitete Militarisierung und Autoritarisierung in einen Zusammenhang mit den wirtschaftlichen Abhängigkeiten von transnational organisierten Industriesektoren stellen (vgl. hierzu Cowen 2014), wie es insbesondere in unseren Beispielen Brasilien und Indien für die tiefere Analyse notwendig wäre. Die staatliche Repression, Polizei- und Militärgewalt gegen Arbeiter:innen stehen hier sowohl in einem engen Zusammenhang mit deren innergesellschaftlicher Marginalisierung auf Basis diskriminierender Zuschreibungen und Ungleichheitsverhältnisse, aber auch mit dominanten Erzählungen globaler Standortwettbewerbe und ungleicher Lieferkettenstrukturen. Beide Komponenten erfordern eine vertiefte und integrierende sozialwissenschaftliche Konfliktforschung.

## Footnotes

1 Die Recherche innerhalb einschlägiger Zeitschriften der Friedens- und Konfliktforschung im deutsch- und englischsprachigen Raum zeigt, dass Lieferketten zwar behandelt werden (vgl. Choi und Smith 2010; Drost et al. 2014), allerdings eher ein Randthema sind.

2 Die sozialwissenschaftliche Auseinandersetzung mit globalen Lieferketten hat außerhalb der Betriebswirtschaftslehre diverse Konzepte geprägt, etwa Global Commodity Chains, Global Value Chains und Global Production Networks. Wir setzen uns in diesem Beitrag nicht mit den Unterschieden dieser Konzepte auseinander, vgl. hierzu Gibbon et al. (2008); Henderson et al. (2002).

3 Ausnahmen sind etwa Bair und Werner (2011); LeBaron und Lister (2015); Levy (2008); Zajak und Scheper (2019).
